# Impact of Heat Stress on Selected Parameters of Robotic Milking

**DOI:** 10.3390/ani11113114

**Published:** 2021-10-30

**Authors:** Roman Gálik, Angélique Lűttmerding, Štefan Boďo, Ivana Knížková, Petr Kunc

**Affiliations:** 1 Institute of Agricultural Engineering, Transport and Bioenergetics, Faculty of Engineering, Slovak University of Agriculture in Nitra, Tr. A. Hlinku 2, 949 76 Nitra, Slovakia; roman.galik@uniag.sk (R.G.); stefan.bodo@uniag.sk (Š.B.); 2 Livestock Technology and Management, Institute of Animal Science, Přátelství 815, Uhříněves, 104 00 Praha, Czech Republic; knizkova.ivana@vuzv.cz (I.K.); kunc.petr@vuzv.cz (P.K.)

**Keywords:** temperature-humidity index, automatic milking system, dairy cow, average milk speed, maximum milk speed

## Abstract

**Simple Summary:**

The aim of this study was to verify the effect of temperature stress on dairy cows milked in an automatic milking system in the temperate climate of central Europe. The study showed that there was not a decrease in the daily milk yield per milking if the temperature-humidity index value was lower than 68. This was accomplished with the dairy cows having an average daily milk yield of 29 kg to 31 kg. A decrease of the daily milk yield per cow was recorded if the temperature-humidity index value was greater than 72. A higher average milk speed, as well as a higher maximum milk speed, may occur with a temperature-humidity index value higher than 68.

**Abstract:**

The values of the temperature-humidity index and its influence on the performance parameters of dairy cows were monitored on four farms located in the southern part of the central Slovakia during a period of three years. The observed parameters included: the milk yield per cow per day, average milk speed and maximum milk speed. The thermal-humidity index was calculated based on a formula. The individual periods were divided according to the achieved THI. The results of dairy cows with a milk yield of 29 kg to 31 kg show that there is not a decrease in the milk yield per milking if the THI value is lower than 68. It was also found that there was a decrease in the milk yield per dairy cow in the robotic milking parlor for a THI value greater than 72. The influence of a THI value higher than 68 in these dairy cows results in a higher average milk speed, as well as a higher maximum milk speed. These two parameters are not yet in the main area of research interest. This study enriches the area with new knowledge, according to which dairy cows can show thermal stress by increasing the milk speed as well as the maximum milk speed.

## 1. Introduction

Automatic milking systems (AMS) have been used in dairy farms more and more over the past two decades. The system gives an opportunity for cattle to decide when to be milked [1]. The other advantages include the reduction of the workload for the staff on the dairy farm, along with the possibility of milking more than twice a day without higher labor costs. However, the AMS does not only include its own robotic milking also represents a completely new management on dairy farms [2,3,4].

However, the expected positive effects of this type of milking may be disturbed by inappropriate microclimatic and macroclimatic conditions [5,6]. In particular, heat stress does significantly affect the welfare of cattle with all its negative effects, such as a reduced milk yield, reduced feed intake, increased water consumption, changes in the milk composition, impaired reproductive performance, etc. [7,8,9,10,11].

The Thermal Humidity Index (THI) is frequently used to describe the heat load. It is a very important indicator of the quality of the barn environment. It does combine the effect of the air temperature and the relative humidity [9,12,13,14,15,16]. The THI model does not take into account other environmental factors, such as the air velocity or the solar radiation intensity; the THI is still considered to be an excellent indicator of stressful climatic conditions [9,14].

A high THI value does negatively affect the milk yield of dairy cows [9,12,14,17]. A value of THI above 72 is considered to be a critical value [6]. A decline in the milk yield was also noted two days before reaching the limit values of THI [18,19]. A decrease in the milk yield was found at a four-day interval when the THI value was above 72 [20]. The daily milk production began to decline between THI values of 65 to 76 [21]. A THI value of 68 does adversely affect the dairy cow’s organism [22,23]. The limit for dairy cows producing more than 35 kg of milk per day is a THI value of 68 [24].

The aim of this study was to analyze the impact of THI on selected parameters of robotic milking.

## 2. Materials and Methods

### 2.1. Farms and Animals

The study was carried out on four dairy farms (A, B, C, D) with the AMS in the Slovak Republic. The herds comprised lactating dairy cows of the Holstein breed after the first calving, as well as older ones. The dairy cows were kept in a loose housing with cubicle beds & bedding with free access to all milking robots and were fed the same total mixed ration. Two types of the AMS were used on these farms—the Lely Astronaut A3 and the Lely Astronaut A4 (Lely, Maassluis, The Netherlands). Table 1 shows the details of these farms.

### 2.2. Data Collection

The study lasted between 2015, 2016 and 2017. The monitored parameters were: the milk yield per cow per day (kg/day), average milk speed (kg/min) and maximum milk speed (kg/min). These data were obtained from the farm reports (AMS reports) of the selected farms.

Next, the air temperature and the relative humidity were continuously measured throughout the study to calculate the THI. The air temperature and its relative humidity were recorded by Comet COMMETER D3120 recorders (COMET SYSTEM, s.r.o., Roznov pod Radhostem, the Czech Republic). These measuring devices were placed in the barn in such a way that the cows did not have access to these devices.

The THI was calculated based on the values of the air temperature and the relative humidity. The following formula was used [25]:

(1) $$\mathrm{THI}=\mathrm{Tdb}-\left[0.55-\left(0.55\times \frac{\mathrm{RH}}{100}\right)\right]\times \left(\mathrm{Tdb}-58\right)$$

where:

Tdb = air temperature, °F;

RH = relative humidity, %.

Individual THI values were classified into the following zones [9,18], listed in Table 2.

We monitored the data from the three days before reaching the second zone of the THI (68–71), followed by the data from the days when the THI was above 68 and the data from the one day after the THI fell below 68.

### 2.3. Data Analysis

In the evaluation we only included the data from the summer period—June, July and August—in each monitored year. The farms were evaluated individually. The data from the three days before reaching the second zone of the THI (68–71) were monitored, followed by the data from the days when the THI was above 68 and the data from the one day after the THI fell below 68.

Table 3 lists the details of the zones of evaluation we used to describe the graphs.

The evaluation included the following effects: the effect of the THI on the milk yield per cow per day (kg/day), the effect of the THI on the average milk speed (kg/min) and the effect of the THI on the maximum milk speed (kg/min).

### 2.4. Statistical Analysis

The obtained data were initially processed in Microsoft Office Excel (Microsoft, Redmond, the United States of America). The program Statistica 7 CZ (TIBCO, Palo Alto, UNITED STATES OF AMERICA) was used for the statistical evaluation of the results and for creating the graphs. The data were expressed as means ± SD (standard deviation). A one-way ANOVA and the Tukey’s HSD post hoc test were utilized. A 95% confidence interval was selected (*p* < 0.05).

## 3. Results and Discussion

Table 4 shows the most number of days with a THI value greater than 68. They were recorded in the first year and measured on all four farms. The most days with a THI of 72–79 were recorded on farm A. The most days with a THI value greater than 68 were recorded on farm C in the first year. In total, the most days with a THI value greater than 68 were recorded on farm A during the total 125 days in the summer period.

Table 5 shows the results of the descriptive statistics of the data from all four farms. There was only one milking robot on farm A, and on farms B, C, and D there were four milking robots. Only the data from June, July and August during the three years were statistically processed. Using these data, the impact of THI on individual parameters was monitored and statistically evaluated.

The highest average value of the milk yield per cow per day was found on farm D, with 31.27 kg. The highest average value of the milk speed was found on farm D, with 2.93 kg/min. The highest average value of the maximum milk speed was found on farm D, with 4.13 kg/min. The highest value of the THI was found on farm A, with a THI value of 70.11.

**Figure 1:**
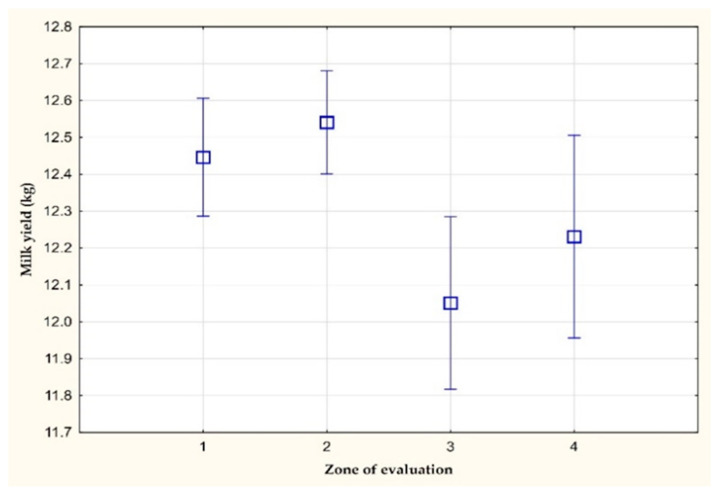
The amount of milk yield per milking (kg) on farm B.

Figure 1 shows a decrease in the daily milk yield, specifically on farm B. With a THI value greater than 72 (3rd zone of evaluation), a statistically significant (*p* < 0.05) decrease of 0.40 kg and 0.49 kg was found in the milk yield, respectively, when compared to the 1st or 2nd zone of evaluation. After the return of a THI value below 68 (4th zone of evaluation), there was a statistically insignificant increase in the daily milk yield (from 12.05 kg to 12.23 kg).

**Figure 2:**
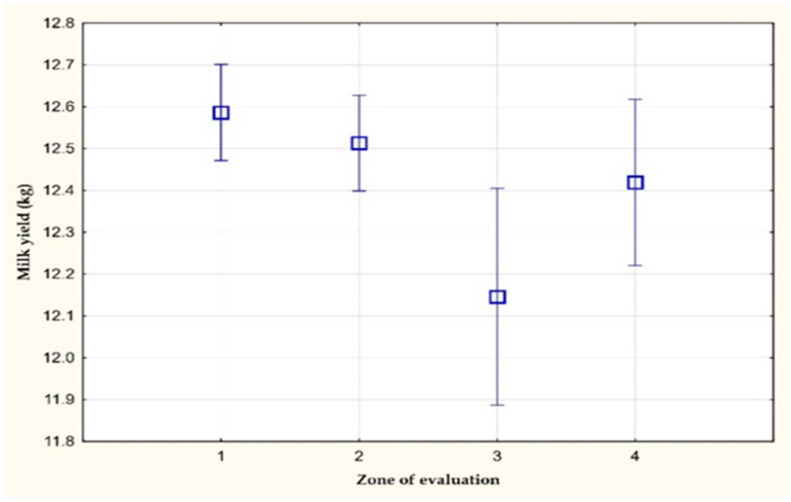
The amount of milk yield per milking (kg) on farm D.

Figure 2 shows the average values of the daily milk yield on farm D. A statistically significant difference (*p* < 0.05) in the milk yield was recorded between the 1st and 3rd zone of evaluation. The decrease in the milk yield was 0.44 kg/day. The value of the daily milk yield in the 4th zone of evaluation did not reach the original levels, which were from before the effect of heat stress. This result is in agreement with the research of [9,12,14,17]. The authors reported that a high THI value negatively affected the milk production of dairy cows. The daily milk yield did not return to its original level from before the effect of heat stress, which was related to the physiological reactions of cattle to heat stress [26,27].

**Figure 3:**
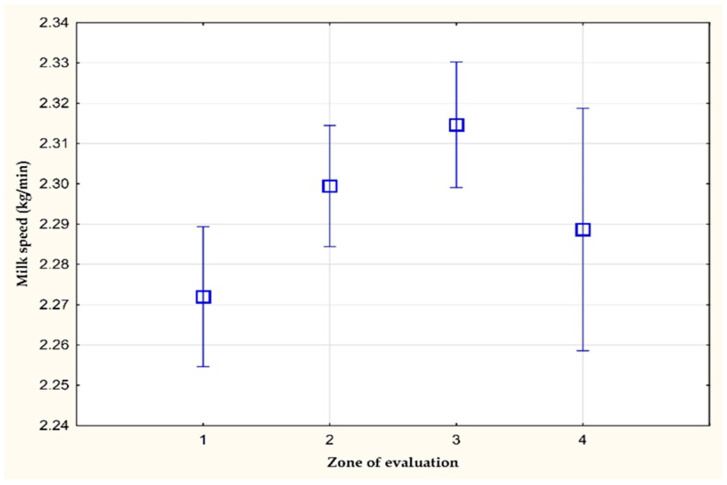
The average milk speed (kg/min) on farm C.

**Figure 4:**
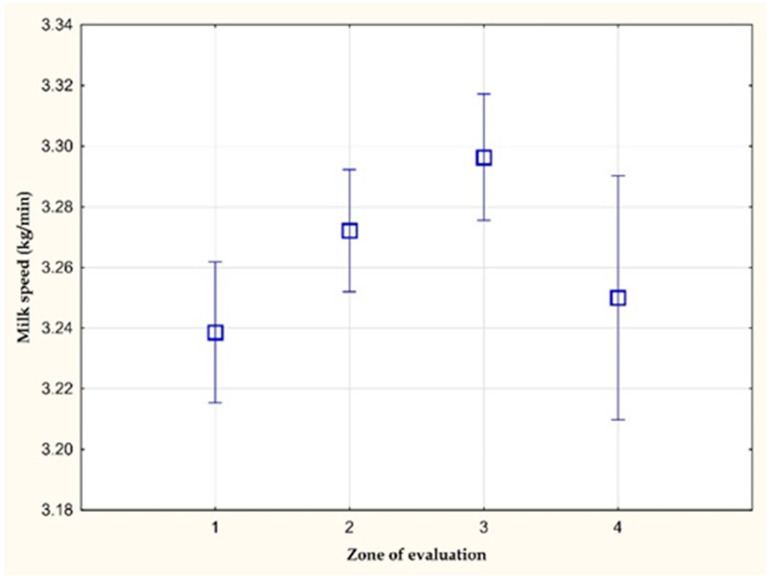
The maximum milk speed (kg/min) on farm C.

Figure 3 and Figure 4 show the average values of the average milk speed and maximum milk speed on farm C. A statistically significant difference (*p* < 0.05) was observed in the average milk speed between the values of the 1st and 3rd zone of evaluation. The average milk speed in the stress-free zone (1st zone of evaluation) was 0.04 kg/min lower than in the zone with a THI value greater than 72 (3rd zone of evaluation). The other differences were not statistically significant. However, a statistically significant difference (*p* < 0.05) in the mean values of the maximum milk speed was found between the values of the 1st and 3rd zone of evaluation (0.06 kg/min).

**Figure 5:**
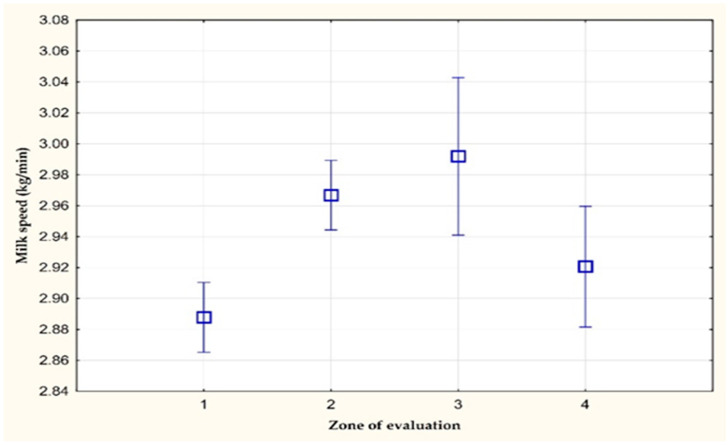
The average milk speed (kg/min) on farm D.

**Figure 6:**
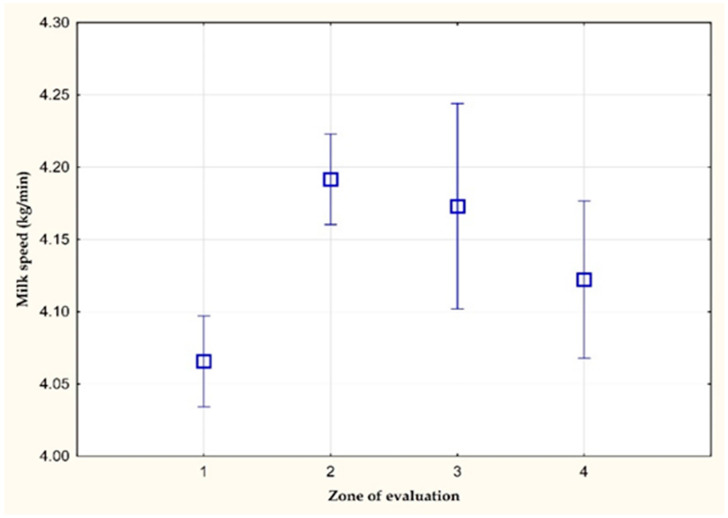
The maximum milk speed (kg/min) on farm D.

Figure 5 and Figure 6 show the average values of the average milk speed and maximum milk speed on farm D. In the case of this farm, a statistically significant difference was found between the values of the 1st and 2nd zone of evaluation (0.08 kg/min), and also between the values of the 1st and 3rd zone of evaluation (0.11 kg/min). The other differences were not statistically significant. However, a statistically significant difference (*p* < 0.05) in the mean values of the maximum milk speed was found between the values of the 1st and 2nd zone of evaluation (0.13 kg/min) and between the 1st and 3rd zone of evaluation (0.11 kg/min). The other differences were not statistically significant.

These results show that when a dairy cow is under heat stress, the animal tries to eject milk more quickly [26]. This is probably due to the fact that the cow is trying to get rid of the accumulated heat in the form of milk. This is in agreement with the claims of [19,28,29,30,31], who state that the metabolic heat output increases with the amount of milk, and so that the cows try to get rid of this metabolic heat. The genetic selection for the milk speed is feasible, and the existence of a correlation structure between the milk speed and milk yield, however, necessitates a selection strategy to increase the milk speed with no repercussion on the genetic merit of the milk yield [32,33]. However, heat stress can negatively affect these milking parameters. In the 4th zone of evaluation, the original level was not reached, which is again related to the reactions of cattle to the heat [26,27].

## 4. Conclusions

Despite the fact that the dairy cows on our farms had the same feed ration, plenty of drinking water and the same standard of animal welfare all the time, we can conclude that a reduced milk yield, increased average milk speed and increased maximum milk speed are related to THI. The results showed that at a THI value higher than 72, the daily milk yield was negatively affected. A higher average milk speed as well as a higher maximum milk speed may occur at a THI value higher than 68. It was found that the day after the decrease of the THI value under 68, all of the monitored parameters improved. However, these parameters did not reach the original levels, which were before the effect of the THI. From these results it can be concluded that the effect of heat stress lasts significantly longer in dairy cows, even if the temperature-humidity conditions return to optimal values.

## Figures and Tables

**Table 1:** Description of the dairy farms.

Farm	Type of the Automatic Milking System	Number of the Automatic Milking System on the Farm	Number of Dairy Cows	Average Annual Performance per Dairy Cow (kg)
A	Lely Astronaut A3	1	58	6517.00
B	Lely Astronaut A4	4	288	8717.56
C	Lely Astronaut A4	4	295	7500.00
D	Lely Astronaut A4	4	303	8710.25

**Table 2:** Overview of the zones calculated according to the temperature-humidity index.

Number of the Zone	Range of the Values in the Zone	Classification of the Zone
1	64–67	stress-free zone
2	68–71	very mild stress zone
3	72–79	moderate stress zone
4	80–89	severe stress zone
5	90–98	extreme stress zone
6	99–104	death zone

**Table 3:** Zones of evaluation.

Number of the Zone	Description of the Zone
1.	3 days before the zone of a very mild stress
2.	very mild stress (68–71)
3.	moderate stress zone (72–79)
4.	the day after the decrease of THI under 68

**Table 4:** Number of days with the highest temperature-humidity index value.

Farm	Year	Days with Temperature-Humidity Index Value in Range of 68–71	Days with Temperature-Humidity Index Value in Range of 72–79
A	1	22	31
	2	19	12
	3	25	16
B	1	26	12
	2	17	2
	3	16	7
C	1	26	29
	2	18	12
D	1	26	4
	2	12	2
	3	10	3

**Table 5:** Results of the descriptive statistics of all four farms.

Farm	Parameter	Average	Minimum	Maximum	Standard Deviation
A	Milk yield per cow per day (kg)	19.09	15.3	24.6	1.61
	Average milk speed (kg/min)	1.72	1.4	2.1	0.18
	Maximum milk speed (kg/min)	2.81	2.6	3.2	0.13
	THI	70.11	59.55	78.29	3.9
B	Milk yield per cow per day (kg)	28.96	18.8	38.3	3.23
	Average milk speed (kg/min)	2.81	2.3	3.3	0.17
	Maximum milk speed (kg/min)	3.93	3.3	4.5	0.21
	THI	68.04	58.74	75.84	3.58
C	Milk yield per cow per day (kg)	23.34	13.3	27.2	1.65
	Average milk speed (kg/min)	2.3	2.0	2.6	0.1
	Maximum milk speed (kg/min)	3.3	3.0	3.8	0.14
	THI	69.6	59.31	76.85	3.95
D	Milk yield per cow per day (kg)	31.27	24.3	38.3	2.46
	Average milk speed (kg/min)	2.93	2.3	3.3	0.16
	Maximum milk speed (kg/min)	4.13	3.4	4.6	0.23
	THI	67.55	59.83	74.86	3.36

## Data Availability

The datasets generated during and analysed during this study are not publicly available, but are available from the corresponding author on a reasonable request.
